# Risk factors for low back pain among elementary school students in western Iran using penalized logistic regression

**DOI:** 10.4178/epih.e2020039

**Published:** 2020-06-02

**Authors:** Forouzan Rezapur-Shahkolai, Elham Gheysvandi, Leili Tapak, Iman Dianat, Akram Karimi-Shahanjarini, Rashid Heidarimoghadam

**Affiliations:** 1 Department of Public Health, School of Public Health, Hamadan University of Medical Sciences, Hamadan, Iran; 2 Social Determinants of Health Research Center, Hamadan University of Medical Sciences, Hamadan, Iran; 3 Research Center for Health Sciences, Hamadan University of Medical Sciences, Hamadan, Iran; 4 Department of Biostatistics, School of Public Health, Hamadan University of Medical Sciences, Hamadan, Iran; 5 Modeling of Non-Communicable Diseases Research Center, Hamadan University of Medical Sciences, Hamadan, Iran; 6 Department of Occupational Health and Ergonomics, Faculty of Health, Tabriz University of Medical Sciences, Tabriz, Iran; 7 Department of Ergonomics, School of Public Health, Hamadan University of Medical Sciences, Hamadan, Iran

**Keywords:** Health promotion, Prevalence, Posture, Musculoskeletal pain, Child, Adolescent

## Abstract

**OBJECTIVES:**

This study investigated the prevalence of low back pain (LBP) and its risk factors among elementary-school students.

**METHODS:**

In this cross-sectional study, 693 elementary students from Hamadan city, western Iran, were selected by multistage stratified cluster sampling. Data were collected through interviews using questionnaires. Posture and psychosocial elements were assessed using the observational Rapid Upper Limb Assessment (RULA) checklist and the standard Strengths and Difficulties Questionnaire, respectively. Penalized logistic regression with the group smoothly-clipped absolute deviation regularization method was used for variable selection and data analysis (α=0.05). The chi-square test was also used.

**RESULTS:**

In total, 26.6% of the students (7-12 years old) reported LBP in the last month. Older age (odds ratio [OR], 3.08; 95% confidence interval [CI], 1.80 to 5.26), watching TV for more than 3 hours a day (OR, 2.62; 95% CI, 1.46 to 4.68), very short seat backrests (OR, 3.08; 95% CI, 1.61 to 5.90), excessively curved seat backrests (OR, 4.36; 95% CI, 2.08 to 9.13), very short desks (OR, 3.44; 95% CI, 1.61 to 7.35), a family history of LBP (OR, 2.49; 95% CI, 1.58 to 3.91), carrying a school bag on one shoulder (OR, 1.91; 95% CI, 1.03 to 3.54), and RULA scores of 3 (OR, 2.26; 95% CI, 1.13 to 4.50) or 4 (OR, 2.85; 95% CI, 1.37 to 5.91) were associated with LBP.

**CONCLUSIONS:**

A high prevalence of LBP was found among elementary-school students. This study underscores the importance of recognizing vulnerable children and teenagers and developing interventional health promotion programs to prevent LBP based on an appropriate consideration of its contributory factors.

## INTRODUCTION

According to the 2017 Global Burden of Disease study, low back pain (LBP) was ranked first among health issues in terms of years lived with disability (YLDs) [1]. It is also the second highest cause of YLDs among 15-19-year-old, accounting for more YLDs than other health problems, such as asthma, alcohol consumption, drug use, and road traffic injury [2]. LBP is a prevalent condition among school-age students, but it has not received sufficient attention [3]. In developing countries such as Thailand, Zimbabwe, and Bosnia and Herzegovina, it was reported that 14.9%, 42.9%, and 16.0% of the student population suffered from back pain, respectively [4-6]. In 2015, it was found that 34.3% of Iranian students in the 11-14-year-old age group experienced LBP [7].

LBP is defined as pain between the costal margins and inferior gluteal folds, which is usually accompanied by painful motor restrictions and may be related with referral pain to the foot, but is not a result of direct trauma or systemic diseases such as neoplasms, infections, peripheral arterial disease, metabolic conditions, or endocrine malfunction [8]. LBP can considerably restrict daily activities, such as attending school and gym or taking part in sports [9]. As LBP in adults may originate from childhood and adolescence experiences [10], recognizing it at young ages is necessary for designing effective prevention and management strategies [11].

Although previous studies have investigated a broad range of variables, including biological parameters (such as weight, muscle strength, or ergonomics), psychosocial factors (such as family and societal relationships or satisfaction with school), lifestyle-related variables (such as physical activities [PAs], watching TV, or computer use), and posture, as potential causative factors of LBP in children and teenagers [11-16], inconsistent results have been reported regarding these risk factors and their relationships with LBP in children and teenagers. Furthermore, studies that incorporate all the above factors in a single analysis are quite limited [6,7,17]. In addition, few studies have addressed the prevalence and risk factors of LBP in students at younger ages [6,16].

Therefore, this study, as a part of broader research project on musculoskeletal problems, from which results about neck and shoulder pain have previously been reported [18], was conducted with the purpose of assessing LBP prevalence and its potential risk factors among elementary-school students.

## MATERIALS AND METHODS

### Study design and setting

This cross-sectional study was conducted in Hamadan from early January 2018 to late March 2018. Hamadan is a city in the west of Iran with a population of 573,449 based on the Health Center statistics from 2018.

### Sampling/participants

The sample included 693 elementary-school students (7-12 years old) and their parents. Multistage stratified cluster sampling was conducted. Hamadan has 2 educational areas (strata), according to the divisions of the Hamadan Department of Education. In the first phase of sampling, a list was provided by the Department of Education and then 13 schools (clusters) were selected randomly from the above areas based on socioeconomic conditions. In the second phase, all 6 grades of each selected elementary-school (1 class from each grade) were considered, and finally students were selected randomly from each class.

### Measures

The questionnaires developed by Dianat et al. [7] and Hatami et al. [19] were used to gather information about demographic variables, leisure time (including age, sex, PA, cell phone use, computer use, computer games, watching TV), family history of LBP, school-related factors (information about convenience and comfort of the school furniture based on a modification of the Chair Feature Checklist), classroom design and amount of homework, and some information about the type of school bag used, how students carried their school bags, and the length of time spent carrying the school bag. These questionnaires have been shown to be adequately reliable and valid. Minor revisions were made according to experts’ opinions to adapt the items of the questionnaires for the population of the present study. The questionnaires were tested in a pilot study of 60 students. According to their comments, partial revisions were made to a number of items in the questionnaire to improve transparency and understandability. Additionally, the test-retest approach was used to assess the reliability of the questionnaire items using the kappa coefficient (ranging from 0.73 to 0.95) and the intraclass correlation coefficient (ranging from 0.85 to 0.98).

The Rapid Upper Limb Assessment (RULA) checklist, which is an observational method for measuring the risk of upper limb musculoskeletal disorders, was used to observe students’ postures. On this checklist, the observations were recorded as numerical scores. These scores were converted into final scores using the RULA scoring matrix, according to which the priority level of corrective steps was determined (level 1: a score of 1 or 2, indicating that a posture is acceptable if not maintained for a long period of time; level 2: a score of 3 or 4, indicating that further investigation is needed and changes may be required; level 3, a score of 5 or 6, indicating that an investigation is needed and changes are required soon; and level 4, a score of 7 or higher, indicating that further investigation and immediate changes are required) [20]. The questionnaire’s reliability and validity were confirmed by Dianat & Salimi [21]. Each student’s posture was recorded by an observer using the RULA checklist to control for inter-observer variability. Inter-rater reliability was assessed at 2 different times, using a pilot study among 60 students in the current study. The Cronbach alpha coefficients for the upper arm, lower arm, wrist, neck, trunk, and leg were 0.79, 0.82, 0.78, 0.83, 0.84, and 0.86, respectively, indicating an acceptable level of reliability.

The standard Strengths and Difficulties Questionnaire (SDQ) was used to measure psychosocial variables. This questionnaire uses a 5-factor structure to measure behavioral-emotional problems and prosocial behaviors in 4-17-year-old children. It also involves 4 subscales related to difficulties (emotional problems, conduct problems, hyperactivity, and peer problems) along with a strengths measure (prosocial behaviors). This 25-item questionnaire was filled out by students’ parents. Each question was scored on a 3-point Likert scale as 0 (not true), 1 (somewhat true), and 2 (certainly true). Each subscale had 5 questions and the theoretical range of the strengths and difficulties scores was 0-10 and 0-40, respectively. The total difficulty score was obtained from the 4 subscales related to difficulties. According to the obtained scores, each subscale is divided into 3 categories (normal, borderline, and abnormal) [22]. The reliability and validity of the questionnaire have been reported to be acceptable [23].

A pre-shaded manikin picture to show areas of pain was used to measure the prevalence of LBP, with an accompanying question reading “Have you experienced pain or discomfort in your back area for a day or more during the last month? (responses: yes/no)” [7,24]. Pain intensity was measured using a visual analogue scale graded from 0 (no pain) to 10 (worst pain) [25]. All the questionnaires were filled out during interviews conducted by the principal researcher and the students’ posture was recorded by an ergonomic specialist.

Finally, the weights of students and their school bags were measured using a digital electronic scale (Beurer, Ulm, Germany) with a sensitivity of 100 g. Each participant’s standing height was measured using a portable stature meter (Yongkang Putai Hardware Factory, Yongkang, China). Each participant’s body mass index (BMI, kg/m^2^) was calculated and classified according to the World Health Organization (WHO) growth reference charts (2007) into 3 categories: healthy weight (healthy weight (5th-85th percentile), underweight (< 5th percentile), and overweight (≥ 85th percentile) [26].

LBP was considered as the outcome of interest (a binary response variable). The independent/exposure variables that were included in the analytical models were the students’ demographic and PA/leisure activity characteristics (age, sex, BMI, hours per week playing sports, hours per day using a mobile/tablet, hours per day using a computer, hours per day playing games, hours per day watching TV); classroom furniture/layout design (seat height, seat backrest height, seat backrest inclination, seat backrest curvature, seat depth, seat width, desk height, seat pan inclination, seatto-blackboard distance, classroom teacher placement, viewing the blackboard); amount of homework, position doing homework at home, history of accident or injury related to LBP, family history of LBP, school bag–related variables (type and weight of school bag, duration and method of carrying the school bag, method of travel to/from school); psychosocial factors (prosocial behavior, emotional symptoms, conduct problems, hyperactivity, peer problems, total difficulties), and RULA score.

### Statistical analysis

Penalized logistic regression was utilized to select important correlates of LBP. We used the group smoothly-clipped absolute deviation (SCAD) penalty in the logistic regression model to select correlates and to measure the associations between LBP and demographic characteristics and PA/leisure activity, school-related factors, psychosocial factors, and RULA scores. Briefly, this model is a shrinkage regression model that imposes an L1 penalty on the regression coefficients. The chi-square test also was used to analyze the data in this study.

Stepwise logistic regression is the usual approach to select variables associated with a binary response; nevertheless, it suffers from some disadvantages, including instability of the selected variables and vulnerability to overfitting. Recently, penalized regression models, in which a penalty term is attached to the likelihood function and estimation and variable selection is done simultaneously, have shown promising results. Among the various penalties, the use of SCAD has been well established as a way to obtain reliable results [27].

The SCAD penalty is defined as follows:

$$\rho_{SCAD}\;(\beta ;\;\lambda ,\;\gamma )\;=\;\left\{\begin{array}{l} \frac{\begin{array}{c} \lambda \left|\beta \right|,\;\;\;\;\;\;\;\;\;\;\;\;\;\;\;\;\;\;\;\;\;\;\;\;\;\\ 2\gamma \lambda \left|\beta \right|\;-\;(\beta^{2}\;+\;\lambda^{2}) \end{array}}{2(\gamma -1)}\\ \frac{\lambda^{2}(\gamma^{2}\;-\;1)}{2(\gamma -1)} \end{array}\right.,\;\begin{array}{c} if\;\left|\beta \right|\leq \lambda ,\;\;\;\;\;\;\;\;\;\;\;\\ if\;\lambda \;<\;\left|\beta \right|\;\leq \gamma \lambda ,\\ if\;\left|\beta \right|\;>\;\gamma \lambda .\;\;\;\;\;\;\; \end{array}\;\;\;$$

In the above equation λ is the tuning parameter and its optimum value should be obtained through cross-validation. In this study, we used a 10-fold cross-validation strategy. The value of λ with the smallest Bayesian information criterion was chosen as the optimum value. This method was repeated 1,000 times and the estimated coefficients were averaged over all repetitions. To estimate the standard errors of the coefficients, a bootstrap strategy was used with 1,000 replications. Therefore, 1,000 samples (with replacement) were selected from the original data and then the standard errors of the coefficients were computed to calculate 2-sided p-values. A significance level of 0.05 was adopted for all statistical analyses.

The adequacy of the final model was checked using the Hosmer & Lemeshow test, in which a p-value greater than 0.05 indicates no evidence of poor fit [29].

The area under the receiver operating characteristic curve (AUC) was also used to check the predictive accuracy of the model. To do this, the data were divided randomly into 2 subsets (training and testing sets). The model was fitted to the training data and the AUC was obtained for the testing set. AUC values lie between 0.5 and 1, where 0.5 denotes a bad classifier and 1 denotes an excellent classifier.

It should be noted that penalized logistic regression simultaneously considers all independent variables, including potential confounding variables, when selecting variables. Therefore, this model can control for the effect of potential confounding variables. In the current study, demographic characteristics, physical leisure activities, psychosocial factors, and family history of LBP among school students, which could serve as potential confounding variables, were entered into the model as independent variables.

### Ethics statement

This study was approved by the Ethics Committee of Hamadan University of Medical Sciences (approval code: IR.UMSHA.REC. 1396.641). Informed consent was obtained from the students and their parents.

## RESULTS

### Low back pain among participants

In total, 26.6% of the students reported LBP during the last month. The intensity of LBP differed significantly (p=0.001) between males (mean, 1.08; standard deviation [SD], 2.22) and females (mean, 1.79; SD, 3.06). However, there were no significant differences (p=0.106) by sex with regard to absence from school due to LBP.

The mean±SD age of the students’ was 9.70±1.61 years, and their BMI was 17.68±3.57 kg/m^2^ (Table 1).

### Risk factors for low back pain

Using the chi-square test (Table 2), the prevalence of LBP was found to be significantly higher in older students (31.8%, p=0.001) and among female students (29.9%, p<0.05).

According to the penalized logistic regression analysis (Table 3), the following factors had an significant relationship with LBP: older age (odds ratio [OR], 3.08; 95% confidence interval [CI], 1.80 to 5.26; p<0.001), watching TV for more than 3 hours a day (OR, 2.62; 95% CI, 1.46 to 4.68; p<0.001), very short seat backrests (OR, 3.08; 95% CI, 1.61 to 5.90; p<0.001), excessively curved seat backrests (OR, 4.36; 95% CI, 2.08 to 9.13; p<0.001), very short desks (OR, 3.44; 95% CI, 1.61 to 7.35; p<0.001), a family history of LBP (OR, 2.49; 95% CI, 1.58 to 3.91; p<0.001), carrying a school bag on 1 shoulder (OR, 1.91; 95% CI, 1.03 to 3.54; p<0.05), and RULA scores of 3 (OR, 2.26; 95% CI, 1.13 to 4.50; p<0.05) or 4 (OR, 2.85; 95% CI, 1.37 to 5.91; p<0.01).

Model adequacy was confirmed by the Hosmer & Lemeshow test (*χ*^2^= 5.165; degree of freedom [df]= 8; p=0.740). Moreover, the predictive accuracy of the model using the selected variables shown in Table 3 was acceptable (AUC, 0.739) [29].

## DISCUSSION

The results of the current study showed that the prevalence of LBP among elementary students was 26.6%. Older age, watching TV for more than 3 hours a day, a family history of LBP, very short desks, very short seat backrests, excessively curved seat backrests, and carrying a school bag on 1 shoulder were independently related with LBP. RULA scores of 3 and 4 were also independently related with LBP.

In this study, LBP prevalence had a direct relationship with age. This finding aligns with previous studies [15,30-32]. It is likely that students have more homework as they become older and advance to higher grades. Consequently, the weight of their school bags increases, and unsuitable ways of carrying school bags become more common. These findings are supported by a study of school students in Iran that showed less PA among older students [33], although this tendency can be prevented by implementing appropriate interventions [34].

Among the demographic variables, the prevalence of LBP in girls was higher than in boys, which is similar to other studies [7,24]. Boys may have an inclination to underreport or worry less about this problem. Also, girls may engage in less PA than boys, making them more likely to have LBP [33,34].

The following school-related factors were significantly related to LBP: very short seat backrests, excessively curved seat backrests, and very short desks. Sitting at a very short desk causes students to place their head in a forward position, placing the low back under pressure (load), which contributes to LBP [30]. It was found that when furniture 20 centimeters higher than usual was used for a 20-minute reading, lumbar flexion was reduced to a considerable degree (10° compared to 40°) [35]. The backrest being either too low or far back encourages students to lean backwards, so when they simultaneously perform an activity such as writing, the neck area is bent to a remarkable extent [30]. School furniture design contributes to musculoskeletal pain in students [30,36,37]. Two studies in Iran have confirmed the mismatch between school furniture and students’ anthropometric dimensions [38,39]. Some interventions can be recommended to reduce LBP due to improper class furniture, including ergonomic interventions such as proper furniture design (desk height and seat backrest height) in proportion to students’ anthropometric dimensions, training on how to sit properly on classroom furniture, and stretching exercises to prevent sitting for a long time [33,34].

In contrast, individual factors related to PA and leisure time (PA time, cell phone and computer use, and computer games) and BMI were not associated with LBP, which accords with other studies conducted by Diepenmaat et al. [24] in the Netherlands, Murphy et al. [30] in England, and Mohseni-Bandpei et al. [32] and Dianat et al. [7] in Iran.

Watching TV for more than 3 hours a day had a significant relationship with LBP. Studies have shown that watching TV increases the risk of LBP [32,40]. The prevalence of LBP has been reported to exceed more than 50% among those who watched TV for more than 2 hours a day [41]. Similar findings have indicated that the situation is aggravated when watching TV is accompanied by sitting in a relatively static position for a long time, unsuitable sitting postures, and low levels of PA [42,43].

In this study, having a family history of LBP was a predictor of students’ LBP. The presence of such a complaint in children is relevant to the history of similar complaints in their households [30]. The prevalence of LBP among children of whom one parent had this condition was 21%, while it was 24% among children of whom both parents had LBP; in contrast, the prevalence was only 14% among children whose parents were both healthy (with regard to LBP) [42].

RULA scores of 3 and 4 were significantly related with LBP. Studies have reported a significant relationship between bending and rotation of the low back and musculoskeletal pains (including LBP) in school-age children and adolescents. We must consider, however, that those mentioned studies used a different instrument from present study (the portable ergonomic observation method) [44,45]. Students’ undesirable sitting positions in class, as well as listening, reading, and writing in different conditions, result in musculoskeletal pain, especially in their low back area. Unless this condition receives appropriate attention, it may be accompanied by irreparable complications and multiple spinal column problems in the long term [46]. Presenting the necessary instructions and corrective actions for this group of students is recommended to encourage them to adjust their sitting posture and to prevent subsequent related disorders.

In the present study, psychosocial factors were not found to be related to LBP. Previous studies have reported significant relationships of psychological and psychosocial factors with musculoskeletal complaints in children and teenagers [7,16,47]. The reason for this finding may be that the SDQ questionnaires were self-reported by the parents, potentially leading to inaccuracies that could have a negative impact on the study outcomes.

Among the variables related to school bags investigated in this study, only carrying a school bag on 1 shoulder was related to LBP. Studies have shown that heavy school bags and improperly carrying them for long periods of time may adversely affect children’s musculoskeletal system, not only resulting in fatigue and back pain, but also affecting the normal growth of the spine [48,49]. Therefore, recommending a suitable bag weight, producing safe bags, and informing students and their parents of the side effects of having heavy bags and carrying them improperly may be convenient interventions for solving school bag-related LBP problems.

The current study indicated a high prevalence of LBP among elementary-school students and identified some important risk factors, including demographic factors, leisure activities, classroom furniture, manner of carrying a school bag, family history, and improper sitting postures.

LBP may have negative consequences for both the physical performance and the social life of children and adolescents. LBP in childhood and adolescence has also been shown to be associated with LBP in adulthood. Therefore, recognizing these risk factors at an early age can help prevent LBP in adulthood, especially if more effective interventions are developed.

This study simultaneously considered all the factors analyzed in previous studies on LBP, which is a distinctive feature of this study that may be one of its strengths. In addition, few studies have addressed the prevalence and risk factors of LBP in younger students. Another strength of this study is the combined use of individual interviews and posture observations to investigate LBP prevalence and its risk factors among elementary students.

Nonetheless, the study findings should be interpreted in light of some limitations. For instance, the cross-sectional design of the study makes it impossible to infer a causal relationship between the 2 events. Another limitation is related to the validity and accuracy of the data obtained from students’ and parents’ self-reports, which may be accompanied by under-reporting or over-reporting, although this approach is widely used for diagnosing LBPs and problems among children and teenagers [7,24]. It is also worth keeping in mind that not all the data were obtained from self-reports; the data on students’ postures were recorded through observations by an ergonomic specialist and the students’ height and weight and the weight of their bags were directly measured by the principal researcher.

In conclusion, risk factors such as older age, watching TV for more than 3 hours a day, having a family history of LBP, very short seat backrests, excessively curved seat backrests, very short desks, carrying one’s school bag on 1 shoulder and RULA scores of 3 and 4 were associated with LBP, reflecting the multifactorial nature of this condition. Therefore, multifaceted solutions and ways of making effective healthy changes should be considered for maintaining low back health. Recognizing vulnerable children and teenagers, developing interventions such as health education and promotion programs, and involving the educational system, teachers, parents, and the students themselves in interventions targeting health improvement are required. To develop interventional programs, it is indispensable to investigate ways of improving schools and children’s ergonomic conditions, as well as considering ergonomic essentials at home and school for teenagers who are maturing and whose musculoskeletal system is forming.

## Figures and Tables

**Table 1.** Demographic characteristics, variables related to physical/leisure activities, variables related to school bag use, and SDQ scores among the study participants

Variables	n (%)	Mean±SD
Demographic		
Age (yr)		9.70±1.61
Weight (kg)		35.88±16.85
Height (cm)		136±16.32
BMI (kg/m^2^)		17.68±3.57
Physical and leisure activities		
Playing sports (hr/wk)	582 (84.0)	1.70±1.61
Using a mobile device (hr/d)	530 (76.5)	0.81±1.07
Using a computer (hr/d)	133 (19.2)	0.24±0.70
Playing games (hr/d)	174 (25.1)	0.35±0.86
Watching TV (hr/d)	673 (97.2)	2.07±1.58
School bag–related variables		
School bag weight (kg)	-	3.60±0.74
School bag as % of body weight	-	11.4±3.6
Time spent carrying school bag (min)	-	13.7±13.4
Type of school bag		
Backpack	676 (97.5)	-
Other (shoulder bag/wheels)	17 (2.5)	-
Method of carrying one’s school bag		
Both shoulders	589 (85.5)	-
One shoulders	86 (12.4)	-
Other (by hands/wheels)	18 (2.6)	-
Method of travel to/from school		
Car	261 (37.7)	-
Walking	347 (50.1)	-
Other	85 (12.3)	-
SDQ scale		
Total difficulties		
Normal	491 (70.9)	-
Borderline	99 (14.9)	-
Abnormal	103 (14.3)	-
Emotional symptoms		
Normal	515 (74.3)	-
Borderline	57 (8.2)	-
Abnormal	121 (17.5)	-
Conduct problems		
Normal	481 (69.4)	-
Borderline	96 (13.9)	-
Abnormal	116 (16.7)	-
Hyperactivity		
Normal	523 (75.5)	-
Borderline	70 (10.1)	-
Abnormal	100 (14.1)	-
Peer problems		
Normal	397 (57.3)	-
Borderline	144 (20.8)	-
Abnormal	152 (21.9)	-
Prosocial behavior		
Normal	655 (94.5)	-
Borderline	24 (3.5)	-
Abnormal	14 (2.0)	-

SDQ, Strengths and Difficulties Questionnaire; SD, standard deviation; BMI, body mass index.

**Table 2.** Associations between low back pain (LBP) and demographic characteristics

Characteristics	LBP		p-value^1^
	Without	With	
Age (yr)			0.001
<10	247 (79.9)	62 (20.1)	
≥10	262 (68.2)	122 (31.8)	
Sex			0.032
Male	246 (77.4)	72 (22.6)	
Female	263 (70.1)	112 (29.9)	
Body mass index (percentile)			0.880
Healthy weight (5th-85th)	341 (73.0)	126 (27.0)	
Underweight (<5th)	34 (72.3)	13 (27.7)	
Overweight (≥85th)	134 (74.9)	45 (25.1)	

Values are presented as number (%).

1 Using the chi-square test.

**Table 3.** Factors associated with LBP in the penalized logistic regression model

Variables	Total	With LBP	OR (95% CI)	p-value
Age (yr)				
<10	309 (44.6)	62 (20.1)	1.00 (reference)	-
≥10	384 (55.4)	122 (31.8)	3.08 (1.80, 5.26)	<0.001
Seat backrest height				
Just right	263 (38.0)	64 (24.3)	1.00 (reference)	-
Too low	121 (17.4)	44 (36.4)	3.08 (1.61, 5.90)	<0.001
Too high	309 (44.6)	76 (24.6)	1.11 (0.68, 1.82)	0.657
Seat backrest curvature				
Just right	367 (53.0)	76 (20.7)	1.00 (reference)	-
Too flat	78 (11.0)	26 (33.3)	2.33 (0.92, 5.88)	0.074
Too curved	248 (36.0)	82 (33.1)	4.36 (2.08, 9.13)	<0.001
Desk height				
Just right	169 (24.3)	35 (20.7)	1.00 (reference)	-
Too low	131 (19.0)	51 (38.9)	3.44 (1.61, 7.35)	<0.001
Too high	393 (56.7)	98 (24.9)	1.50 (0.77, 2.91)	0.228
Seat-to-(black) board distance				
Just right	146 (21.0)	46 (31.5)	1.00 (reference)	-
Too far	448 (64.8)	110 (24.6)	1.11 (0.53, 2.34)	0.771
Too near	99 (14.2)	28 (28.3)	0.60 (0.31, 1.14)	0.121
History of accident or injury related to LBP				
No	631 (91.0)	174 (27.6)	1.00 (reference)	-
yes	62 (9.0)	10 (16.1)	0.52 (0.22, 1.24)	0.142
Family history of LBP				
No (reference category)	459 (66.2)	101 (22.0)	1.00 (reference)	-
yes	234 (33.8)	83 (35.5)	2.49 (1.58, 3.91)	<0.001
Method of carrying one’s school bag				
Both shoulders	589 (85.0)	144 (24.4)	1.00 (reference)	-
One shoulder	86 (12.4)	34 (39.5)	1.91 (1.03, 3.54)	0.039
Other (by hands/wheels)	18 (2.6)	6 (33.3)	0.91 (0.12, 6.45)	0.927
RULA				
Level 1	99 (14.3)	18 (18.2)	1.00 (reference)	-
Level 2	112 (16.1)	13 (11.6)	0.53 (0.21, 1.33)	0.178
Level 3	301 (43.5)	95 (31.6)	2.26 (1.13, 4.50)	0.020
Level 4	181 (26.1)	58 (32.0)	2.85 (1.37, 5.91)	0.005
Watching TV (hr/d)				
<1	240 (34.7)	60 (25.0)	1.00 (reference)	-
1-3	257 (37.0)	50 (19.5)	0.85 (0.50, 1.44)	0.551
>3	196 (28.3)	74 (37.8)	2.62 (1.46, 4.68)	<0.001
Time spent carrying school bag (min/d)				
≤20	560 (80.9)	140 (25.0)	1.00 (reference)	-
>20	133 (19.1)	44 (33.1)	1.66 (0.95, 2.89)	0.073

Values are presented as number (%). LBP, low back pain; OR, odds ratio; CI, confidence interval; RULA, Rapid Upper Limb Assessment.
